# Cardiac remodeling secondary to chronic volume overload is attenuated by a novel MMP9/2 blocking antibody

**DOI:** 10.1371/journal.pone.0231202

**Published:** 2020-04-09

**Authors:** Lena Cohen, Irit Sagi, Einat Bigelman, Inna Solomonov, Anna Aloshin, Jeremy Ben-Shoshan, Zach Rozenbaum, Gad Keren, Michal Entin-Meer

**Affiliations:** 1 Laboratory of Cardiovascular Research, Tel Aviv Sourasky Medical Center, Tel-Aviv, Israel; 2 Sackler Faculty of Medicine, Tel-Aviv University, Tel-Aviv, Israel; 3 Department of Biological Regulation, Weizmann Institute of Science, Rehovot, Israel; Scuola Superiore Sant'Anna, ITALY

## Abstract

**Objective:**

Monoclonal antibody derivatives are promising drugs for the treatment of various diseases due to their high matrix metalloproteinases (MMP) active site specificity. We studied the effects of a novel antibody, SDS3, which specifically recognizes the mature active site of MMP9/2 during ventricular remodeling progression in a mouse model of chronic volume overload (VO).

**Methods:**

VO was induced by creating an aortocaval fistula (ACF) in 10- to 12-week-old C57BL male mice. The VO-induced mice were treated with either vehicle control (PBS) or with SDS3 twice weekly by intraperitoneal (*ip*) injection. The relative changes in cardiac parameters between baseline (day 1) and end-point (day 30), were evaluated by echocardiography. The effects of SDS3 treatment on cardiac fibrosis, cardiomyocyte volume, and cardiac inflammation were tested by cardiac staining with Masson's trichrome, wheat Germ Agglutinin (WGA), and CD45, respectively. Serum levels of TNFα and IL-6 with and without SDS3 treatment were tested by ELISA.

**Results:**

SDS3 significantly reduced cardiac dilatation, left ventricular (LV) mass, and cardiomyocyte hypertrophy compared to the vehicle treated animals. The antibody also reduced the heart-to-body weight ratio of the ACF animals to values comparable to those of the controls. Interestingly, the SDS3 group underwent significant reduction of cardiac inflammation and pro-inflammatory cytokine production, indicating a regulatory role for MMP9/2 in tissue remodeling, possibly by tumor necrosis factor alpha (TNFα) activation. In addition, significant changes in the expression of proteins related to mitochondrial function were observed in ACF animals, these changes were reversed following treatment with SDS3.

**Conclusion:**

The data suggest that MMP9/2 blockage with SDS3 attenuates myocardial remodeling associated with chronic VO by three potential pathways: downregulating the extracellular matrix proteolytic cleavage, reducing the cardiac inflammatory responses, and preserving the cardiac mitochondrial structure and function.

## Introduction

Valvular heart diseases, congenital heart defects, and arteriovenous malformations lead to the development of sustained mechanical load (volume overload, VO) in the heart. Hearts subjected to this type of overload are challenged with the initiation of progressive left ventricular (LV) remodeling and dilatation, inflammatory cell infiltration, and extracellular matrix (ECM) degradation and reorganization, all of which eventually lead to heart failure with reduced ejection fraction (HFrEF) [1–3]. The prevalence of sustained VO is increasing with the aging of populations, mainly due to the development of valvular diseases including mitral and aortic valve diseases [4]. Unfortunately, despite major advances in cardiac surgery and intervention, [5–8] as well as in medical pharmacological therapy used in the treatment of VO-induced cardiac hypertrophy [9–11], valvular insufficiency, particularly mitral regurgitation, remains in the aging population one of the main etiologies of VO with subsequent HFrEF in the aging population [12]. Therefore, there is great need for novel effective noninvasive therapy that may attenuate the VO-mediated remodeling process and prevent or delay the need for surgery.

During the development of VO, LV dilatation is induced in a sustained manner and is associated with increased expression of pro-inflammatory cytokines and chemokines [13], as well as with partial collagen network disruption by matrix metalloproteinases (MMPs), which represent one of the major key enzymes in ECM turnover and remodeling [14, 15]. MMPs belong to a group of zinc-dependent enzymes that can be subdivided into several subgroups according to their substrate affinity profile, one of which is the gelatinase subgroup that includes MMP2 and MMP9.

Various animal models of myocardial infarction (MI) showed an elevation of MMP2, and MMP9, as well as activation of their endogenous inhibitors, TIMPs -1,-2, and -4 [16]. Additionally, MMP2 and MMP9 (also known as gelatinase A and gelatinase B, respectively) were found to be constitutively overexpressed in models of chronic VO as well as in models of atherosclerosis and cardiomyopathy. Interestingly, targeted deletion of MMP9 in chronic VO-induced heart failure resulted in attenuation of myocardial dysfunction [17].

Positive effects of several broad-spectrum MMP inhibitors was shown in animal models of pressure overload as well as models of VO [18]. However, their efficacy in clinical trials is altogether uncertain, suggesting that a different approach for specific MMP9/2 blockage is warranted. In the current study that involves a murine model of cardiac VO, we utilized a conformational selective unique antibody SDS3, which shows a TIMP–like binding mechanism toward the zinc-protein complex and enzyme surface conformational epitopes of the endogenous-activated form of MMP9 and MMP2 [19]. The results reveal a significant reduction of hypertrophy and ventricular dilatation as well as reduced cardiac inflammation upon antibody treatment. Although early clinical trials targeting MMP2 for improving cardiovascular outcomes have failed [20], the preclinical data presented herein point towards potential therapeutic effects of SDS3 in the attenuation of VO progression and shed light on the putative underlying signaling processes.

## Materials and methods

### Ethics statement

The study was performed following approval of The Animal Care and Use Committee of the Tel-Aviv Sourasky Medical Center (approval number: 16.8.14) which conforms to the policies of the American Heart Association and the Guide for the Care and Use of Laboratory Animals. All mice were kept under optimal conditions (food and water provided ad libitum) at room temperature in a temperature-controlled facility with a 12 h light/dark cycle. Surgeries were performed with isoflurane anesthesia. The animals were monitored 3–4 times per week for potential signs of suffering, weight loss of more than 10% compared to their initial weight, and changes in their behavior, mobility, or body posture. Mice that met one of these latter criteria underwent euthanasia on the same day in order to prevent further suffering. At the experimental endpoint, the mice were euthanized by carbon dioxide (CO2) inhalation. CO2 exposure was carried out using a gradual filling method, which is less likely to cause pain, thus potentially minimizing aversion or distress. A displacement rate of 10–30% of the chamber (volume/min) was performed. After 3–5 minutes, when cessation of any movement was confirmed, the flow rate was raised until death was confirmed.

### Surgical preparation and experimental protocol

VO was induced by an aortocaval shunt operation in 10-12-week-old C57BL/6 mice. Briefly, the mice were anesthetized with 2% isoflurane, after which the aorta and vena cava between the levels of the renal arteries and iliac bifurcation were exposed by dissection of the overlaying adventitia. A needle (30G) was inserted into the exposed abdominal aorta and advanced through the medial wall into the vena cava to create a shunt. The ventral aortic puncture site was immediately sealed with a drop of cyanoacrylate (Crazy Glue) after withdrawal of the needle. Creation of a successful shunt was visualized by the pulsatile flow of oxygenated blood into the vena cava from the abdominal aorta. The abdomen was then closed and the mice were placed on a heating plate until full recovery from anesthesia. Sham animals underwent the same procedure except for the creation of the shunt. In order to alleviate pain, a subcutaneous injection of carprofen (5 mg/kg) was given during the surgery and once daily during the next three days. After the surgery, ACF mice were randomly divided into two groups: one group received phosphate-buffered saline (PBS) solution as vehicle (*n* = 8) and the other group received SDS3 in a concentration of 3 mg/kg (*n* = 10) twice weekly by intraperitoneal injection (more mice were randomized for the SDS3 treatment group since the effect of the antibody on the mice had not yet been determined). Likewise, the sham-operated mice were randomly divided into 2 groups receiving either SDS3 or vehicle (*n* = 6 /arm). The assigned treatment commenced on the day following fistula or sham surgery and continued until completion of the study 4 weeks later. Body weight and heart weight were recorded at the experimental endpoint for determination of the heart/body weight ratio [21, 22]. A transmural section of the heart was fixed in formalin. The remaining LV was snap-frozen in liquid nitrogen and stored at -80°C.

### SDS3 antibody

SDS3 antibody was produced by an innovative immunization strategy that exploits aspects of molecular mimicry to produce inhibitory antibodies with TIMP-like binding mechanisms toward the activated forms of gelatinases-matrix metalloproteinases 2 and 9, as previously reported [27]. Being an IgG1 antibody, the expected elimination half-life of SDS3 is 5 days in mice and 3 weeks in humans. In the current study, an experimental dosage of 3 mg/kg of SDS3 was utilized based on an *in vivo* model for inflammatory bowel disease in which both the preventive dose (1 mg/kg) and the therapeutic dose (5 mg/kg) of SDS3 significantly attenuated the severity of disease progression [19].

### Echocardiography

The mice were anesthetized with 2% isoflurane, and echocardiography was performed using a Vevo 2100 transducer (VisualSonics, Toronto, Canada), while heart rate was kept between 400–500 bpm in accordance with the guidelines for echocardiographic measurements in the murine heart [23]. Measurements were taken on day 1 and four weeks after fistula formation. Two-dimensional-guided M-mode images were recorded in the long-axis view at the left mid-ventricular level. LV end-diastolic and end-systolic volumes (LVEDV and LVESV) were automatically calculated by the Vevo cardiac software using the Simpson’s formula and derived from the dimensions of the LV measured by the operator. The examiner was blinded towards group treatment protocol. The relative changes in cardiac parameters between baseline (day 1) and four weeks' endpoint were calculated according to the following formula:

%ofchange=[(valueonday30)−(valueonday1)]/(valueonday30)*100.

### Quantitative real-time polymerase chain reaction (qRT-PCR) and Western blot analyses in LV lysates

DNA-free total RNA was extracted from LV samples by a standard protocol with an EZ-RNA-total RNA Isolation Kit (Biological Industries, Israel). cDNA synthesis (500 ng) was carried out with the Verso RT-PCR Kit (ABgene, USA) according to the manufacturer’s instructions. qRT-PCR was performed on a Bio-Rad iQ-Cycler using a SYBR Green PCR kit (Invitrogen, Israel). The primer sequences are provided in S1 Data. Western blot was performed according to standard procedure [3] using the following antibodies: mouse monoclonal anti-Cytochrome C (Cyt C) (Cat # ab14734, Abcam, USA), mouse monoclonal anti-Voltage-dependent anion channel (VDAC)1/porin (Cat # ab110325, Abcam, USA), and mouse monoclonal anti- sirtulin (Sirt)3 (Cat # Sc-365175, Santa Cruz Biotechnology, USA), rabbit anti-GAPDH (Cat # 37168, Abcam, USA) followed by goat anti-mouse horseradish peroxidase (Cat # 10015289, Jackson ImmunoResearch Laboratories, Inc. USA) and goat anti-rabbit horseradish peroxidase (Cat # 111035003, Jackson ImmunoResearch Laboratories, Inc. USA)

### Histology and immunofluorescence of LV sections

The animals’ hearts were harvested and fixed in 4% buffered formaldehyde. After paraffin embedding and sectioning, 5-μm sections were stained with Masson’s trichrome according to manufacturer's instructions in order to assess cardiac fibrosis (Sigma, St. Louis, MO, USA). Myocyte cross-sectional areas derived from the middle area of the LV sections were stained with fluorescein-conjugated wheat germ agglutinin (WGA-Alexa Fluor 594, Invitrogen, Carlsbad, CA, USA) and measured by NIH Image J software (National Institutes of Health, Bethesda, MD, USA). At least 50 randomly selected myocytes that had been cut transversely from 3–4 animals/group were measured. The amount of cardiac white blood cells in the LV sections was assessed according to standard procedures using rabbit anti-CD45 polyclonal antibody (Abcam, USA), followed by anti-rabbit 488-labeled secondary antibody (Jackson ImmunoResearch Laboratories, USA) and visualized by confocal microscopy (Zeiss LSM 510, Germany). A total of 16 fields/ animal were analyzed with 4–5 animals/group.

### Quantification of tumor necrosis factor alpha (TNFα) expression

Sera levels of the pro-inflammatory cytokines, TNFα and IL-6, were quantified Quantikine ELISA kits (R&D Systems, Inc., USA) according to the manufacturer’s protocols.

### Zymography of LV samples

Zymography assay using 10% zymogram (gelatin) SDS-PAGE gel (Novex, Life Technologies, USA) was performed on LV samples according to the manufacturer's instructions [24]. Briefly, the experimental tissues were homogenized and diluted in zymogram sample buffer (Bio-Rad, UK), and 30 μg of diluted samples (according to the Bradford quantification method) were loaded onto the gel. Following electrophoresis, the gel was washed with zymogram renaturation buffer (Novex, Life Technologies) and incubated with Zymogram developing buffer (Novex, Life Technologies, USA) overnight at 37°C. Finally, the gel was stained with 0.05% Coomassie brilliant blue G-250 (Bio-Rad, Israel) solution for two hours. After de-staining, gelatinolytic activities were detected as transparent bands against the blue background.

### Cyt C oxidase activity in mitochondrial fractions of LV

Mitochondria/cytosol fractionation was performed using a mitochondria/cytosol fractionation kit according to the manufacturer’s instructions (BioVision, #K256-25, USA). Cytochrome oxidase activity in mitochondrial lysates was measured using the Cyt C oxidase activity colorimetric assay kit according to the manufacturer’s instructions (BioVision, #K287, USA).

### Mass spectra analysis

Samples of LV wall tissue were immediately harvested into liquid nitrogen and stored at -80°C until analysis. Sham (*n* = 3), ACF (*n* = 3) samples were digested by trypsin-EDTA and analyzed by LC-MS/MS on the HFX mass spectrometer (Thermo, USA). The data were analyzed with MaxQuant 1.5.2.8 vs the mouse UniProt database. Peptide‐ and protein‐level false discovery rates (FDRs) were filtered to 1% using the target‐decoy strategy. Protein tables were filtered to include only proteins that were identified in 3 samples in one of the groups. Partek Genomic suite was used for the ANOVA analysis and identification of differentially expressed protein-lists. Presented proteins were those that had a *P*-value of <0.05 with at least a 1.5-fold change. For the expression analysis, we considered only those proteins that were identified with at least two peptides (out of five), each peptide with at least a 95% confidence interval.

### Statistical analysis

SPSS (IBM® SPSS® Statistics; Version 22) was used for statistical analysis. All variables were expressed as means ± standard error of mean (SEM). A Shapiro-Wilk's test (*P* > 0.05) and a visual inspection of histograms, normal Q_Q plots, and box plots showed that the exam scores were approximately normally distributed all groups. The two-tailed Student's *t-*test was used to compare between two groups, and one-way ANOVA followed by Tukey's post-hoc test was used to compare between three groups. *P* < 0.05 was considered statistically significant in all tests. A trend towards significance (T) was considered at 0.05 < *P* <0.1.

## Results

### Model characterization and MMP9/2 expression

**Fig 1:**
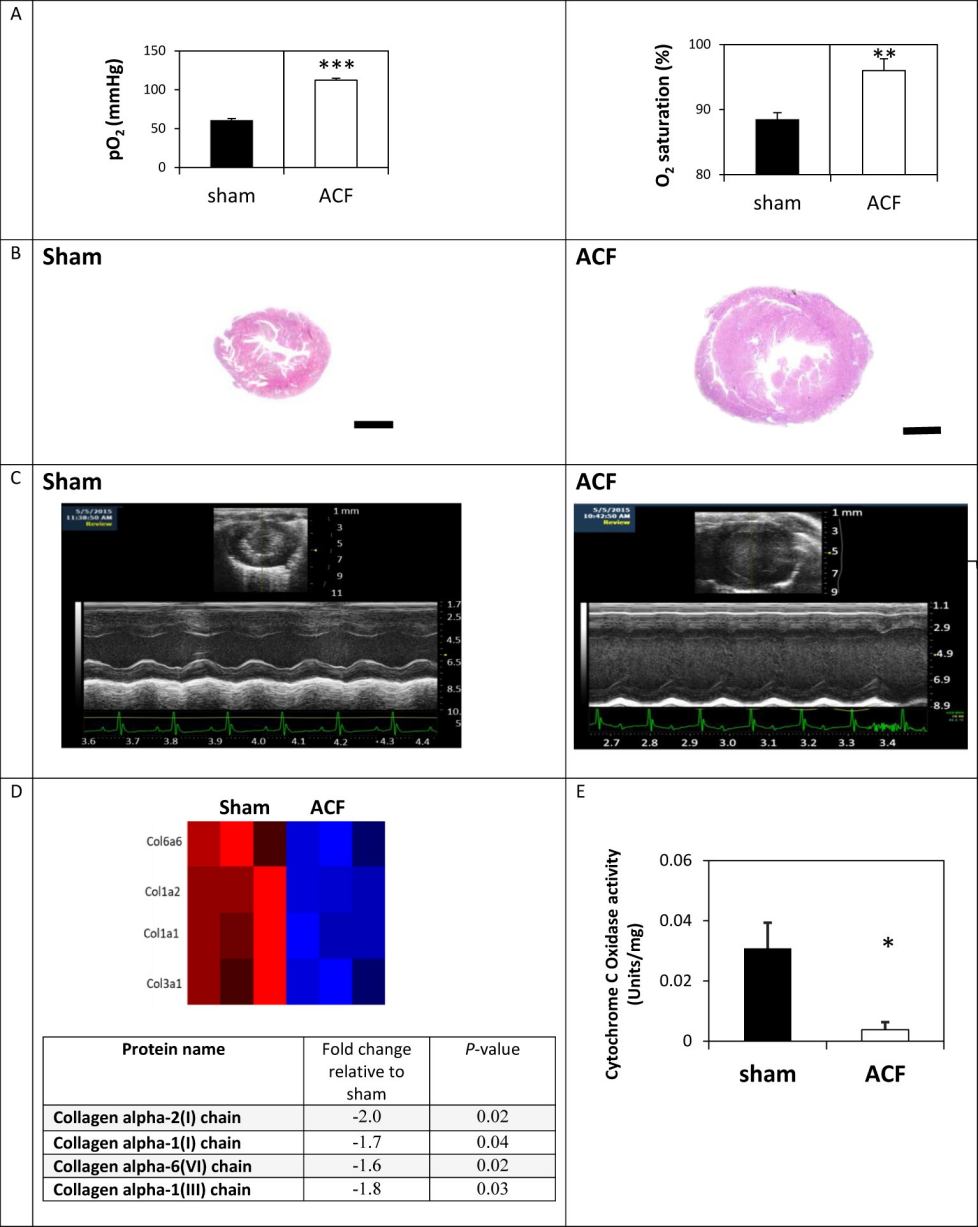
Cardiac dilatation in mice subjected to ACF. (A) Partial oxygen pressure and oxygen saturation at the vena cava one day post-surgery (n = 6 mice per group). (B) Masson's trichrome–stained transverse cardiac sections four weeks post-surgery (representative captures). Scale bar: 1 mm. (C) Representative echocardiographic M-mode images of the same animals. (D) Expression profile of collagen proteins in cardiac sections of sham and ACF animals according to mass spectra analysis. The color scale is ranging from blue (reduced expression) to red (induced expression). (E) Cytochrome C oxidase activity in isolated mitochondria from sham and ACF hearts (n = 5 per group). Data are given as means ± SEM; * *P* < 0.05, **P < 0.01; ***P < 0.001.

**Fig 2:**
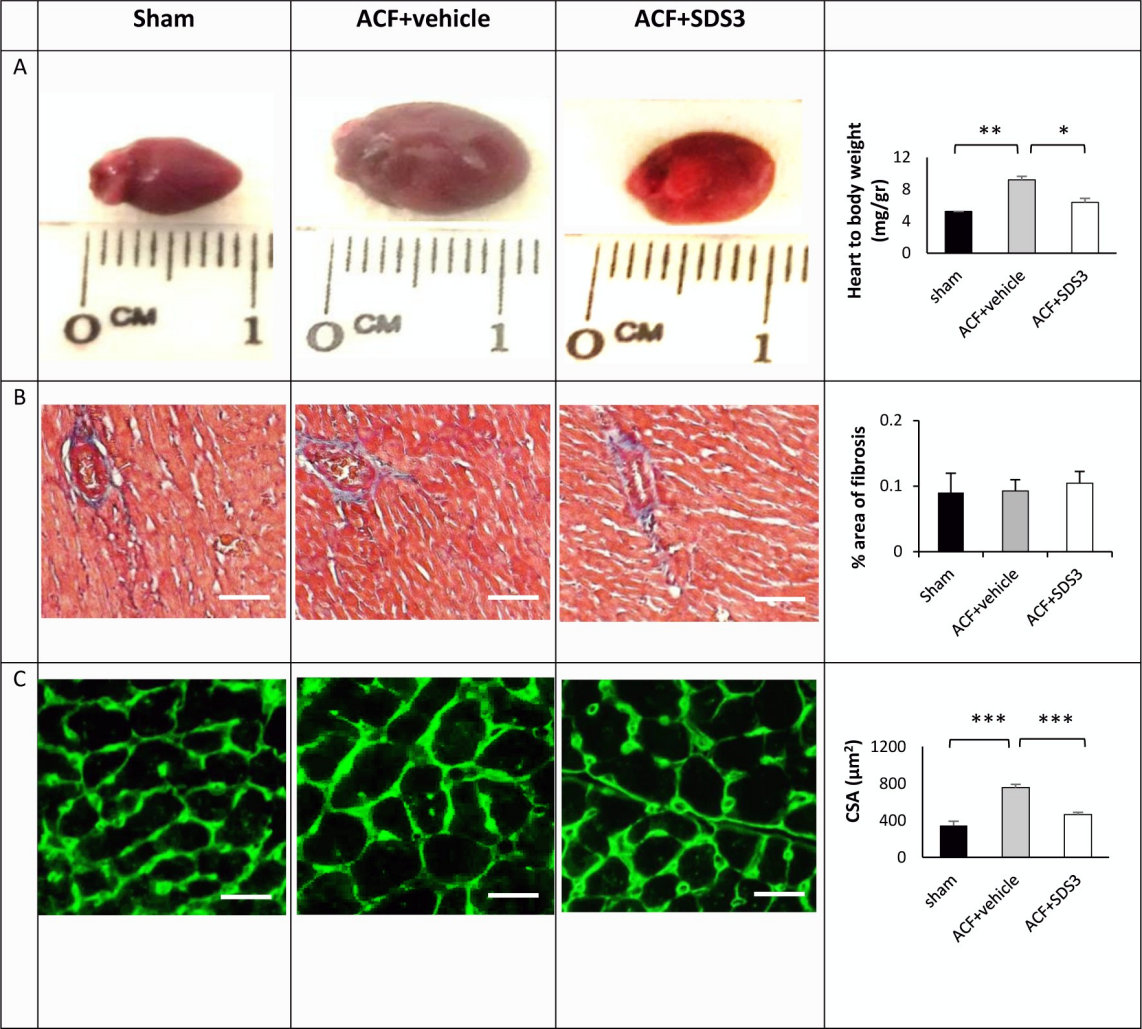
Morphological and cellular changes in ACF animals treated with SDS3 antibody. (A) Left panel: representative captures of heart morphology; Right panel: heart–to-body weight ratio changes in sham (n = 6), PBS-treated ACF (n = 6), and SDS3-treated ACF (*n* = 9). (B) Left panel: representative Masson's trichrome-stained histological sections; Right panel: percentage of fibrosis quantification (n = 5/per group). (C) Left panel: representative pictures illustrating LV myocyte cross-sections stained with WGA. Right panel: cross-sectional area (CSA) quantification (n = 3-4/group). Scale bar: 100 μm.

In order to assess the potential involvement of metalloproteinase activity and ECM turnover, we induced a VO model in mice by means of an aortocaval fistula procedure. We had initially aimed to validate whether the applied methodology involving needle puncture of the abdominal aorta and inferior vena cava could result in a successful shunt. Towards this end, we carried out blood gas tests one day after the surgery to assess the formation of a shunt. The test results demonstrated that the partial oxygen pressure and oxygen saturation at the vena cava were significantly elevated in the ACF group, indicating successful fistula formation (Fig 1A). The model was then applied for four weeks in six test animals and their data were compared to those of six control animals that were subjected to sham operation only. As expected, the VO-induced mice showed elevated LV chamber dimensions and an increased LV mass, as well as an increased ventricle weight-to body weight ratio compared to the sham-operated controls (Figs 1B and 1C and 2A). Also, as expected [25], by employing a mass spectra analysis we have shown that the VO model is associated with a significant collagen degradation (n = 3/group, p < 0.05; Fig 1D).

Since VO induced with acute ACF was reported to cause mitochondrial disorganization and dysfunction [26], herein we wished to assess the effect of chronic ACF on cardiac mitochondrial function. Indeed, the data suggest that prominent mitochondrial damage occurs as evident by the significant loss of Cyt C oxidase activity in the ACF animals compared to the sham controls (0.03 ± 0.008 vs. 0.004 ± 0.002 Units/mg, respectively, *P* = 0.03) (Fig 1E).

### MMP 9/2 antibody treatment

It is well recognized that there is an increase in the expression and activity of MMPs in general and of MMP2 and MMP9 in particular during myocardial remodeling. Therefore, we sought to assess the potential effect of the specific anti-MMP9/2 monoclonal antibody, SDS3, on VO progression in the chronic ACF model. To this end, 18 animals underwent ACF surgery and were randomized to either vehicle (8 mice) or SDS3 treatment (10 mice). One of the 10 SDS3-treated animals died one week post-surgery, while the remaining 17 animals survived until the end of the experiment. We hypothesize that this single fatality was due either to improper *ip* injection that potentially damaged an internal organ or to a fistula which was too large at the outset, so that the animal could not survive it regardless of the treatment. Interestingly, an echocardiographic analysis revealed that the SDS3-treated ACF group underwent a significantly smaller change in cardiac size and mass, including LVEDV, LVESV, and LV mass, compared to the PBS-treated ACF group (*P* < 0.05) (Table 1). The changes in LV diameter were not statistically different between ACF-vehicle to ACF-SDS3 but showed a similar trend. Of note, the volumes of the LV, both diastolic and systolic, were significantly increased in ACF relative to baseline and reduced markedly by treatment with SDS3 compared to the vehicle arm (the difference in LV volume between the two groups was 26 ± 3% for LVEDV and 27 ± 6% for LVESV at the 4-week endpoint [*P* < 0.01 and p < 0.05, respectively]). In line with that finding, the heart weight as measured by echocardiography was significantly reduced from 222 ± 12 mg to 156 ± 13 mg (*P* < 0.05) upon treatment, with no change in the animals’ body weight, pointing to a significant decrease in the heart weight/body weight ratio, as confirmed post-mortem (Fig 2A). No changes in anterior or posterior wall thickness were observed. Of note, LV systolic ejection fraction was still preserved in the ACF group with or without SDS3 treatment, in line with reports demonstrating that LV dysfunction occurs only after >16 weeks post-ACF induction (1). A power analysis confirmed that a total of 14 animals (7/arm) was adequate to determine the magnitude of difference between the two groups (i.e., ACF-vehicle versus ACF-SDS3) in inducing a > 40% increase in LV mass on day 30 relative to day 1 according to the echocardiography measurements. This number of animals was confirmed with confidence intervals (type I/II errors of 0.05 and 0.2, respectively; ClinCalc.com free software). The results indicate a significant attenuation of cardiac dilatation in the SDS3-treated ACF animals compared to the vehicle-treated ACF arm. The mean body weight was similar for the four experimental groups (ACF-vehicle, ACF-SDS3, sham-vehicle, and sham-SDS3). However, the heart weight, as measured during the gross pathologic examination on day 30, was significantly higher in the ACF-vehicle group compared to the SDS3-treated ACF arm (222 ± 12 vs. 156 ± 13 mg; *P* = 0.01), and only slightly but insignificantly higher than that of the sham groups (130 ± 10 and 143 ± 8 mg for the sham-vehicle and sham-SDS3 groups). These data are in line with the LV mass weights calculated by echocardiography which demonstrated a significantly higher degree of change between day 0 and day 30 in the ACF-vehicle group compared to the ACF-SDS3, sham-vehicle, and sham-SDS3 arms (Table 1). Taken together, the heart-to-body weight ratios display substantial differences between the groups, with those of the ACF-vehicle and ACF-SDS3-treated arms being 65% and 16% greater, respectively, than the sham-vehicle group (Fig 2A and Table 1). Of note, SDS3 treatment in all six sham-operated animals did not cause any effect on the cardiac parameters relative to the sham-vehicle arm (Table 1). In addition, no harmful effect was observed on the animals' viability in any of the six SDS3-treated groups.

**Table 1:** SDS3 attenuates ACF-mediated cardiac dilation with no toxic effects on sham-operated controls.

Animal group	ACF+ Vehicle control (n = 8)		ACF+ SDS3 (n = 10)		% of change		Sham+Vehicle control (n = 6)	Sham+SDS3 (n = 6)
Cardiac parameters	day 1	4 weeks after procedure	day 1	4 weeks after procedure	ACF+ vehicle	ACF+SDS3	4 weeks after procedure	4 weeks after procedure
**LVEDD**	3.8±0.1	4.5±0.2	3.7±0.1	4.1±0.1	20±3	10±3	3.75±0.01	3.78±0.1
**LVESD**	2.7±0.1	3.6±0.1	2.7±0.06	3.1±0.1	33±6	15±4	2.8±0.17	2.9±0.17
**LVEDV (μl)**	78±3	133±6	81±3	111±4	40±3	26±3**	71±3	73±3
**LVESV (μl)**	29±2	54±5	27±1	39±3	44±5	27±6*	30±4	33±3
**Ejection fraction (%)**	49±4	45±3	47±4	51±3	1±0.8	7±2	48±4	46±3
**LV mass (mg)**	93.6±5.6	149.0±6.9	80.2±4	108±4.9	62.18±6.3	35.9±10.2*	80±6	86±5
**AW in diastole (mm)**	0.8±0.02	0.9±0.03	0.75±0.2	0.84±0.02	12.2±3	11.8±3	0.7±0.09	0.7±0.01
**AW in systole (mm)**	1.13±0.04	1.1±0.06	1.02±0.03	1.09±0.03	9±4	8±3	0.9±0.1	0.9±0.04
**PW in diastole (mm)**	0.82±0.02	0.9±0.02	0.76±0.01	0.8±0.01	8.5±3	12±2	0.8±0.02	0.8±0.06
**PW in systole (mm)**	1.07±0.03	1.13±0.03	1±0.02	1.1±0.02	8.2±3	8.9±2	1.1±0.04	1.1±0.08
**Ventricle weight (mg)**	NA	222±12	NA	156±13*	NA	NA	130±10	143±8
**Body weight (g)**	23±0.8	24.25±1	23±0.6	24.48±0.5	5.4±0.02	6.2±0.05	24±0.4	23.6±0.5
**Heart rate (bpm)**	415±25	430±19	445±31	431±24	NA	NA	442±40	464±27

Cardiac size and mass parameters of sham and ACF animals with and without treatment with SDS3. % of change in ACF animals was calculated according to the following formula: % change for each parameter = % of change = [(value on day 30)-(value on day 1)]/ (value on day 30) * 100.

LVEDD- LV end-diastolic diameter; LVESD- LV end-systolic diameter; LVEDV- LV end-diastolic volume; LVESV- LV end-systolic volume; AW-anterior wall, PW-posterior wall. Statistical significance in the % of change between ACF+SDS3 and ACF+ vehicle is given as follows:

* *P* < 0.05,

** *P* < 0.01

**Fig 3:**
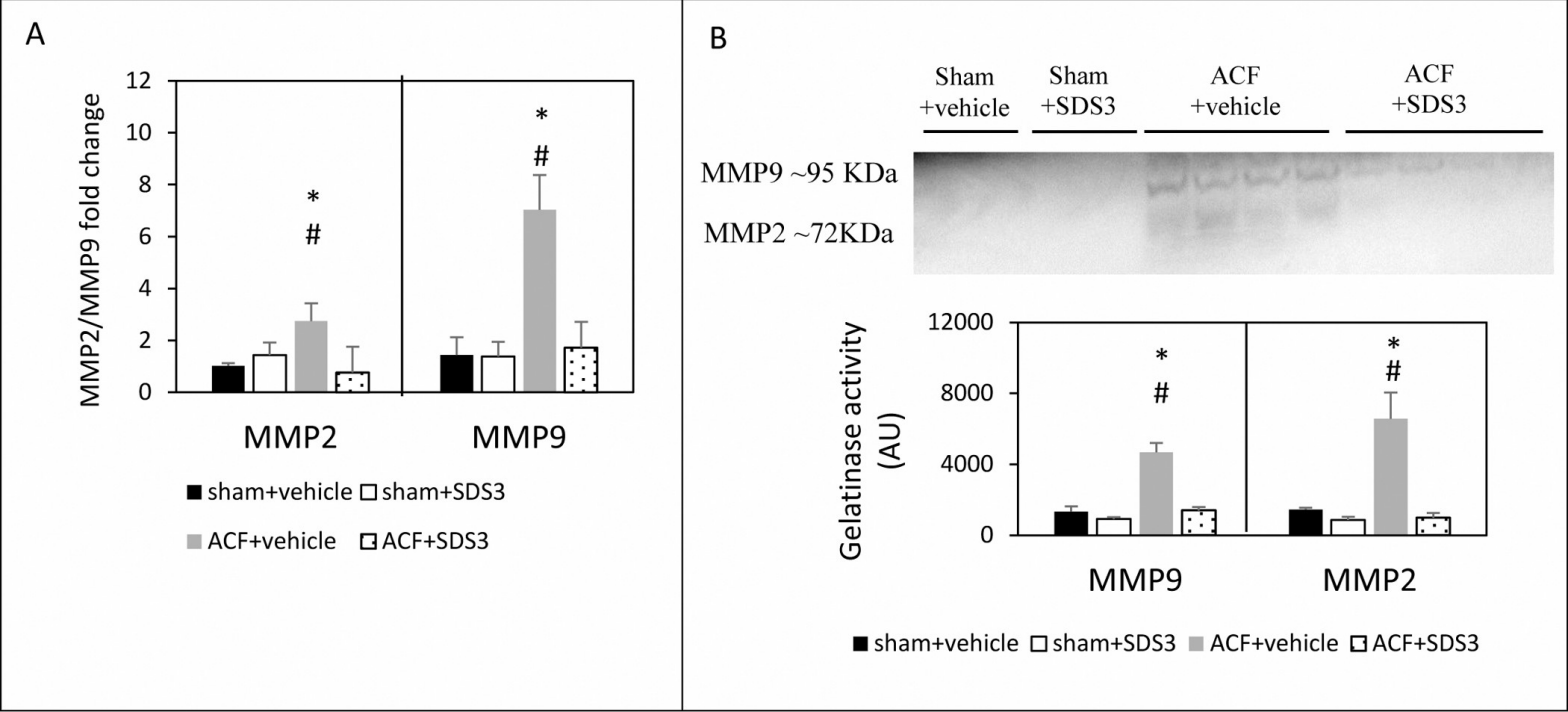
MMP9 and MMP2 expression and gelatinase activity with and without treatment with SDS3. (A) Relative MMP2 and MMP9 mRNA expression (sham-vehicle n = 6, sham-SDS3 n = 6, ACF-vehicle n = 6, ACF-SDS3 n = 8) and (B) gelatinase activity (sham-vehicle n = 2, sham-SDS3 n = 2, ACF-vehicle n = 4, ACF-SDS3 n = 4). Data are given as means ± SEM; ^*^ = *P* < 0.05 relative to sham + vehicle/sham+SDS3, **# =** *P* < 0.05 relative to ACF+SDS3.

Since the potential effects of ECM turnover, particularly those observed in VO progression, are still debatable [27, 28], we applied Masson' trichrome staining of the cardiac sections to assess fibrosis in ACF versus sham following treatment with SDS3. We did not observe any changes in fibrosis between ACF and sham, with or without treatment with SDS3 (Figs 2B and S1), indicating that fibrosis at four weeks of ACF does not represent an underlying mechanism for VO progression. In line with the gross pathology data, LV myocyte size, as determined by WGA histological staining, showed significant differences between the ACF group compared to the sham group (757 ± 35 μm^2^ vs. 345 ± 53 μm^2^; *P* = 0.0003). Interestingly, within the ACF animals, WGA staining in the SDS3-treated group was significantly reduced compared to the vehicle-treated group (466 ± 45 μm^2^ vs; *P* = 0.001), but it was not different from that of the sham-operated controls (*P* > 0.05) (Figs 2C and S1). The beneficial changes in gross pathology of the LV and in the cardiomyocyte size following administration of SDS3 occurred concomitantly with the reduction of MMP9/2 expression and gelatinase activity observed in the cardiac lysates of the ACF-SDS3 mice relative to the ACF-vehicle controls. Indeed, MMP9 mRNA expression was significantly higher in ACF group compared to the sham+vehicle group (7 ± 1 arbitrary units- AU vs. 1.6 ± 0.6 AU, respectively, *P* = 0.03) and then lowered following treatment with SDS3 (1.7 ± 0.7 AU, *P* = 0.05) to levels comparable to those observed in sham animals (*P* > 0.05). Likewise, MMP2 mRNA expression was significantly higher in ACF group relative to the sham + vehicle group (2.7 ± 0.2 AU vs. 1.0 ± 0.6 AU, respectively, *P* = 0.05) and then lowered following treatment with SDS3 (0.7 ± 0.1 AU, *P* = 0.04) to values comparable to those observed in sham animals (*P* > 0.05) (Fig 3A). In line with the mRNA expression data, MMP9/2 activity was significantly increased in ACF compared to sham mice (MMP9: 4655 ± 543 AU in ACF+ vehicle vs. 1334 ± 290 AU in sham+ vehicle, *P* = 0.04; MMP2: 6562 ± 1485 AU in ACF+ vehicle vs. 1454 ± 92 in sham+ vehicle, *P* = 0.05). Following treatment with SDS3, MMP9/2 activity in the ACF mice was reduced (MMP9: 1399 ± 190 AU, *P* = 0.04; MMP2: 975 ± 274 AU, *P* = 0.05) to levels which were similar to those of sham operated controls (*P* > 0.05) (Fig 3B). Of note, no significant changes in MMP9/2 expression and gelatinase activity were documented in the SDS-treated compared to the vehicle treated-sham mice, suggesting no effect of SDS3 on MMP9/2 expression and activity in the normal heart (Fig 3). The data suggest that SDS3 treatment can attenuate the major ECM turnover processes taking place during VO disease progression.

### MMP9/2 as inflammatory mediators in chronic volume overload

**Fig 4:**
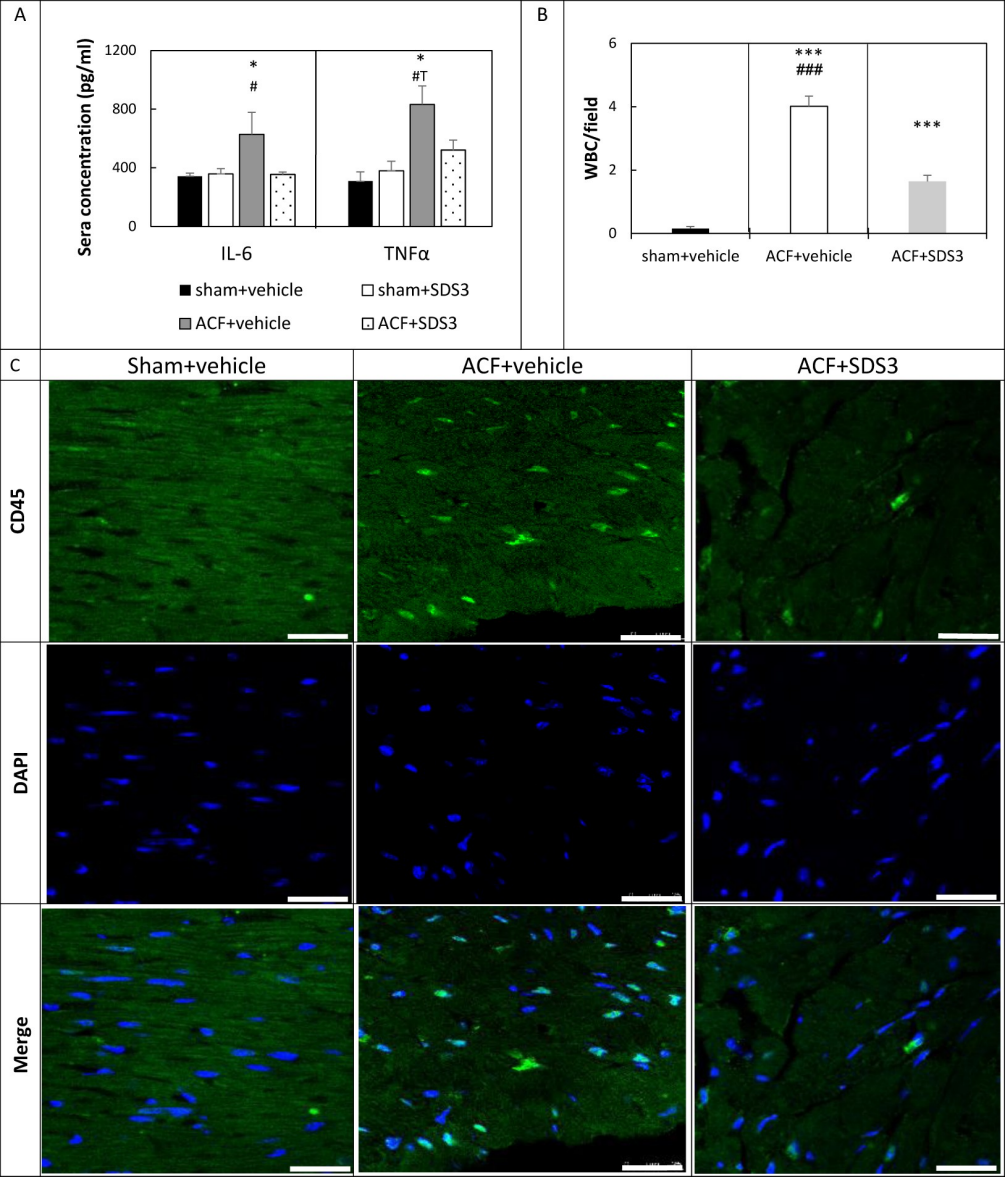
MMP9/2-mediated inflammation is reduced by SDS3. (A) TNFα and IL-6 concentration in sera of sham-vehicle (n = 6), sham-SDS3 (n = 6), ACF -vehicle (n = 6), and ACF-SDS3 (n = 8). Data are given as means ± SEM; * = *P* < 0.05 (relative to both sham+ vehicle and sham+SDS3), **#** = *P* < 0.05 (relative to ACF+SDS3). (B) Mean total number of white blood cells (WBC) per field (16 fields/ animal) in sham, vehicle-treated ACF and SDS3-treated ACF groups. *** P < 0.001 (relative to sham), ### P < 0.001 (relative to ACF+SDS3). (C) Representative immunofluorescent images of CD45 expression (green) in LV sections. Scale bar -25μm. Dapi staining of nuclei is represented in blue.

Heart failure development is associated with an elevated expression of multiple pro-inflammatory cytokines, and both IL-6 and TNFα are associated with left ventricular remodeling and myocyte hypertrophy. TNFα is one of the most important mediators of inflammation which are substantially increased upon remodeling of the heart and its levels may be reduced upon inhibition with MMP blocker [13]. Equally interesting, SDS3 treatment of DSS-induced colitis mice significantly lowered IL-6 levels [19]. Therefore, we sought to assess TNFα and IL-6 levels in the sera of ACF animals with and without treatment of SDS3. As expected, the sera levels of TNFα were markedly elevated in the ACF animals compared to sham + vehicle control group (831 ± 134 pg/ml vs. 308 ± 63 pg/ml, *P* = 0.03) and lowered following treatment with SDS3 (520 ± 70 pg/ml) with a trend towards significance relative to ACF (*P =* 0.06). The reduced TNFα in the ACF+SDS3 animals was statistically similar to that observed in both sham groups (*P* > 0.05) (Fig 4A). Similarly, the levels of IL-6 were significantly increased in the ACF group relative to the sham + vehicle group (628 ± 140 pg/ml vs. 343 ± 20 pg/ml, respectively, *P* = 0.04), and then lowered upon treatment with SDS3 (353 ± 18, *P* = 0.05) down to values which are comparable to those of sham animals (*P* > 0.05) (Fig 4A). Of note, SDS3 treatment did not significantly change the TNFα and IL-6 levels in the sham + SDS3 arm compared to the sham + vehicle arm (*P* > 0.05).

Having documented an elevated expression of the inflammatory mediator TNFα at 4 weeks after fistula formation, we sought to explore whether the number of infiltrating leukocytes is modified upon VO development and subsequent treatment with SDS3.

Quantification of the immunofluorescence staining with CD45 antibody demonstrated that the total number of white blood cells was substantially elevated in the LV sections of the ACF group (4.01 ± 0.3 cells/field in the ACF group versus 0.1 ± 0.06 cells/field in the sham group, *P* < 0.001). SDS3 treatment reduced leucocyte numbers to an intermediate level between sham and ACF (1.6 ± 0.2 cells/field, *P* < 0.001, Fig 4B and 4C). Altogether, the results indicate that SDS3 antibody successfully attenuates the potentially destructive inflammatory processes mediated by the MMP9/2 gelatinase activity.

### Effect of MMP9/2 inhibition on the expression of key mitochondrial proteins

**Fig 5:**
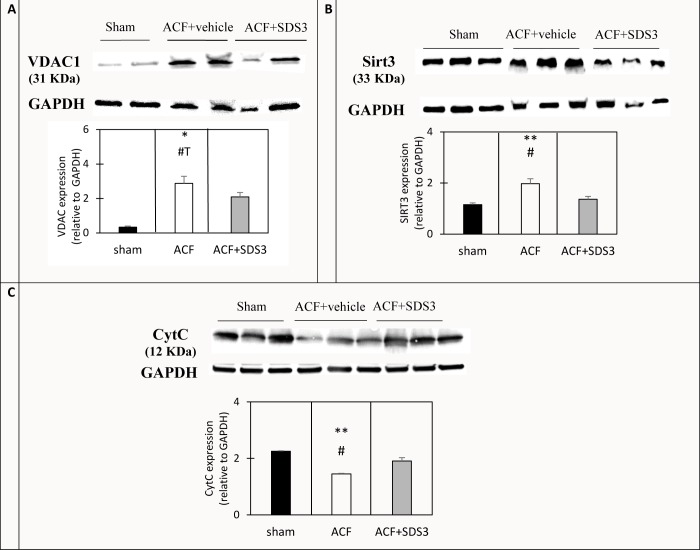
Representative Western blot analysis of mitochondrial proteins in LV tissue homogenate (B) and mitochondrial-fraction lysate (A, C). The lower panel displays quantification of the mitochondrial proteins. *P < 0.05, **P < 0.01- relative to sham, # *P* < 0.05, #T = 0.05 < *P* < 0.1 relative to ACF+SDS3. n = 2–3 per group.

We had shown a clear reduction in Cyt C oxidase activity that suggested more mitochondrial damage in the ACF group compared to the sham group (Fig 1D). Therefore, we now sought to evaluate the degree of mitochondrial integrity in chronic ACF with and without treatment with SDS3 by assessing the expression level of three key mitochondria proteins: Cyt C, VDAC1, and Sirt3, by Western blot analysis. First, we tested the expression of Cyt C, which is normally located in the mitochondrial intermembrane space and represents a key component of the mitochondrial electron transport chain [29]. Cyt C could thus serve as a marker for structural damage to the mitochondrial membrane. The results demonstrated a significant reduction in Cyt C expression in the LV mitochondrial fractions of the ACF animals, presumably due to its leakage from the mitochondria to the cytosol in the ACF animals compared to the sham animals (2.2 ± 0.08 AU vs. 1.4 ± 0.2 AU, respectively, *P* = 0.004, Fig 5C), pointing to substantial membrane damage in the ACF cardiac mitochondria. Interestingly, SDS3 treatment resulted in an increased Cyt C expression in the mitochondrial fractions to an intermediate level between the vehicle-treated ACF group and the sham control group (1.9 ± 0.1 AU, *P* = 0.07, Fig 5C), suggesting a potential partial protective effect of SDS3 on the mitochondrial membrane.

We also tested the expression levels of the voltage-dependent anion channel or the mitochondrial porin (VDAC) protein that is one of the components of the mitochondrial permeability transition pore. Its elevated expression was previously reported in acute VO [48]. Likewise, the data presented herein show that VDAC expression was eight times higher in ACF compared to sham (2.8 ± 0.07 AU vs. 0.3 ± 0.02 AU, respectively, *P* = 0.02, Fig 5A). The VDAC levels in the SDS3-treated group were lower compared to the ACF group (2 ± 0.2 AU, *P* = 0.07). Mitochondrial Sirt3 has been reported to play important roles in maintaining basal ATP levels and regulating ATP production, which is required by the healthy heart. Sirt3 was significantly upregulated in the ACF hearts and downregulated upon treatment with SDS3 to expression levels similar to those monitored in untreated sham samples (1.15 ± 0.06 AU in the sham group compared to 1.9 ± 0.1 AU in ACF, *P* = 0.01 vs 1.3 ± 0.3 AU in the SDS-treated group, *P* = 0.03, Fig 5B). Altogether, the observed changes in the expression pattern of the mitochondrial proteins (Fig 5) as well as the Cyt C oxidase activity (Fig 1E) in ACF versus sham suggest major mitochondrial damage that could be reversed, at least in part, by the SDS3 antibody (Fig 5).

## Discussion

Surgical repair or replacement of the mitral valve is currently the only recommended therapy for severe primary mitral regurgitation, one of the leading causes of cardiac VO. The chronic elevation of wall stress mediated by VO leads to structural remodeling of the muscular, vascular, and ECM components of the myocardium. These changes are initially compensatory, but have detrimental effects in the long term, which ultimately result in heart failure [30, 31]. Although alterations in LV size induced by cardiac VO may represent an important compensatory mechanism for maintaining cardiac function, it is nevertheless well recognized that prolonged cardiac remodeling should be prevented in order to attenuate progression to HFrEF. It is currently accepted that an increase in the expression and proteolytic activity of cardiac MMPs in general, and of MMP2 and MMP9 in particular, is particularly relevant in myocardial remodeling by enhancing partial degradation of the ECM [18, 32]. In line with our data, previous reports have shown that sustained VO is characterized by the loss of collagen which surrounds the cardiomyocytes [25]. This interstitial collagen degradation leads to extensive myocardial hypertrophy and marked ventricular dilatation through a process of myocyte and myofiber slippage along with cardiomyocyte elongation. The process is accompanied with minimal perivascular fibrosis which is apparent only in the later stages of VO progression [18]. Previous reports have shown that treatment with the broad MMP inhibitor PD166793 largely prevented the adverse remodeling in a rat ACF model with no effect on myocardial fibrosis [18]. At the same time, however, selective small molecule blockers to MMP9, which have been designed based on motifs of the active site, were found to be susceptible to active proteolysis in vivo, thus limiting their suitability for application in the clinical setting [33, 34]. In addition, due to the high structural homology shared by various MMPs, the majority of the developed compounds are broad-spectrum blockers that affect the proteolytic activity of other MMPs. To date, none of the broad-range systemic MMP inhibitors have successfully passed clinical trials, and it appears that designing in vivo efficacious highly selective MMP blockers is not a small matter. Therefore, the use of selective function-blocking monoclonal antibodies directed against the activated forms of MMP9/2, such as SDS3, is of substantial advantage [19]. The data presented herein in the murine model for ACF which leads to chronic VO strongly suggest that the SDS3 antibody attenuates ventricular dilatation and hypertrophy, which is typically induced by chronic VO.

The results presented herein suggest three potential mechanisms by which SDS3 may beneficially affect cardiac remodeling. The first and direct mechanism involves attenuation of the ECM proteolytic activity of MMP9/2 as evidenced by zymography assay. The reduced proteolytic activity of MMP9/2 is known to attenuate the disruption of the collagen fibers and thereby to prevent subsequent cardiomyocyte cell slippage and changes in the ventricular geometry [35]. The other two mechanisms described below may indirectly stem from the preserved ECM state mediated by the specific MMP9/2 inhibition.

The second effect of SDS3 involves the attenuation of cardiac inflammatory responses, as evidenced by reduced—expression of the pro-inflammatory cytokines TNF and IL-6 in cardiac sections following treatment with SDS3, as well as by the lower numbers of infiltrating leukocytes observed upon treatment. Accumulating reports suggest that TNFα is released from cell membrane-anchored precursors by proteolytic cleavage, and that broad-spectrum inhibitors of MMPs prevent the processing of pro-TNFα substrates to yield mature TNFα [36, 37]. Moreover, it is recognized that inflammation initiates cardiac remodeling, [38, 39] and that MMP9 plays important roles in immune cell function in various diseases, including arthritis, diabetes, and cancer, as well as in cardiovascular pathologies, such as hypertension, atherosclerosis, myocardial infarction, and cardiac VO [40]. In line with our data, it has been reported that MMP9-deficient mice in an MI model displayed a reduced rupture rate and attenuated ventricular dilatation, which was associated with reduced macrophage infiltration [41, 42]. Interestingly, it has been suggested that TNFα mediates a major myocardial inflammatory response to mechanical stretch in early VO [43]. In line with this observation, in another MI animal model, the authors demonstrated that an excess of TNFα in the myocardium was directly related to increased production of local MMP9 and MMP2 [44, 45]. Moreover, the addition of a broad-spectrum MMP blocker prevented the increased levels of TNFα in a rat ACF model [13], shedding light on the potential effects of MMPs on TNFα-mediated cardiac inflammation.

The third potential favoring effect of SDS3 may involve the preservation of the structure of the cardiomyocytes' mitochondrial membranes, thus allowing normal cellular respiration. Indeed, it was recently reported by Dell’Italia et *al*. that rats inflicted with acute or chronic ACF demonstrate a progressive loss of organization in cardiomyocyte cytoskeletal-mitochondrial architecture, mitochondrial matrix swelling, and cristae fragmentation [46]. Likewise, the data presented herein point to an abnormal respiratory electron transport chain in chronic VO, as evidenced by the decreased mitochondrial Cyt C oxidase activity in ACF vs. sham animals. Treatment with broad-spectrum MMP -inhibitor reportedly prevented the dissolution of interstitial collagen and disarrangement of subsarcolemmal mitochondria in rats induced with acute VO [26]. In the current chronic VO model, we observed increased expression of Cyt C and reduced expression of VDAC in the mitochondrial fraction, as well as an induced expression of Sirt3 in LV homogenates of ACF animals following treatment with SDS3. VDAC is known to control metabolic cross-talk between mitochondria and the rest of the cell by allowing the entry of metabolites, such as pyruvate, succinate, nucleotides, and other compounds into the mitochondria [47]. It was recently demonstrated that a panel of apoptotic inducers upregulated VDAC1 expression levels in a Ca^2+^dependent manner, resulting in VDAC oligomerization, Cyt C release form mitochondria to the cytosol, and subsequent apoptosis [48]. It has also been proposed that the overexpression of VDAC1 in cardiovascular diseases, as well as in other diseases, is associated with mitochondrial dysfunction [49]. Increased VDAC levels were found to be associated with mitochondrial swelling and outer mitochondrial membrane rupture in a model of hepatic inflammation [50]. We therefore anticipate that the elevated expression of Cyt C together with reduced expression of VDAC that had been observed upon treatment with SDS3 is associated with improved organization and function of the cardiac mitochondria. The increased expression of Sirt3, a recognized regulator of ATP production, may represent an attempt to preserve the energetic state of the cardiomyocytes during the compensatory phase of VO disease progression. Indeed, Sirt3 was reported to attenuate cardiac hypertrophy and dysfunction induced by transverse aortic constriction through regulation of the acetylation of mitochondrial proteins [51, 52]. One potential mechanism for the protective role of Sirt3 may be mediated through activation of the peroxisome proliferator-activated receptor γ coactivator 1α, which takes part in mitochondrial biogenesis, as suggested by others [53], although the potential association of Sirt3 with cardiac hypertrophy remains to be clarified.

**Fig 6:**
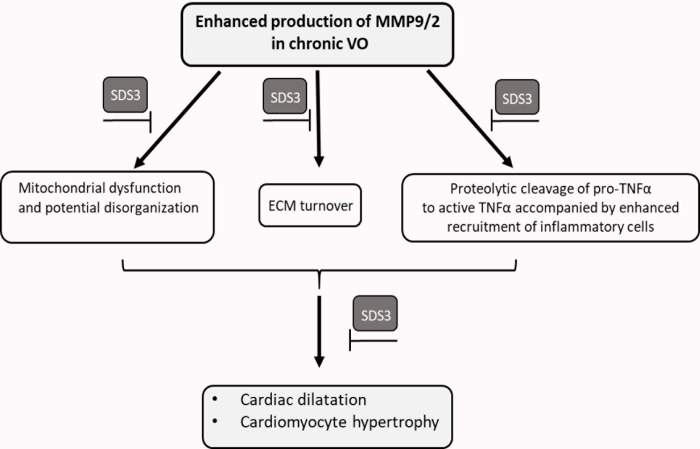
Schematic overview of the potential MMP-9/2 activity in chronic VO which is specifically attenuated by SDS3.

A schematic diagram for the proposed three underlying mechanisms of the induced activity of MMP9/2 and the specific inhibition of that activity by SDS3 in chronic ACF is given in Fig 6. It is of great interest to further study the potential beneficial effects of SDS3 in an acute MI model which is vastly recognized to involve major changes in the ECM structure as well as stormy immune response and metabolic changes. The acute MI model will thus be in the focus of our next study with SDS3.

## Study limitations

The echocardiographic measurements were taken under heart rate of 400 bpm which may limit the accuracy of the measurements. In addition, the direct effect of SDS3 was measured relative to vehicle control but not to IgG isotype control. This group of antibody treatment control will be included in our future experimental procedures. Moreover, further studies should be conducted in order to validate the roles that MMP9/2 play in the disorganization and dysfunction of cardiac mitochondria in chronic VO and the potential beneficial effects of SDS3 on these processes. Special attention should be given to differentiate between subsarcolemmal versus interfibrillar mitochondria with regard to MMP9/2 activity in chronic VO. Furthermore, since MMP9 and MMP2 possess both deleterious and beneficial effects, depending upon the time of the progression of the cardiovascular disease, the exact dose and timing for SDS3 administration should be carefully monitored in order to optimize the treatment and attenuate progression to HFrEF.

## Conclusion

The chronic stage of VO-mediated ECM disruption is associated with MMP9/2 activation, cardiomyocyte hypertrophy, decreased function of the cardiac mitochondria, and increased inflammation. Specific MMP9/2 inhibition by the SDS3 antibody attenuates cardiac hypertrophy, cardiomyocyte remodeling, leucocyte infiltration and mitochondrial damage. Although MMP9/2 represents only a small fraction of the extensive family of MMPs, its specific inhibition affects the key processes that culminate in the attenuation of VO progression to dilated cardiomyopathy and its associated HFrEF.

## Supporting information

S1 Fig (A) Left panel: representative pictures illustrating LV myocyte cross-sections stained with WGA. Right panel: cross-sectional area (CSA) quantification (n = 3-4/group). Scale bar—100 μm (B) Left panel: Representative Masson's trichrome-stained histological sections; Right panel: Percentage of fibrosis quantification (n = 5/per group). (C) Left panel: Representative immunofluorescent images of CD45 expression (green) in LV sections. Dapi staining of nuclei is represented in blue. Right panel: Mean total number of white blood cells per field. Scale bar -25μm.
(TIF)

S1 Data Primer sequences used for RT-PCR. (PDF)
