# Correction: Cardiac remodeling secondary to chronic volume overload is attenuated by a novel MMP9/2 blocking antibody

**DOI:** 10.1371/journal.pone.0241419

**Published:** 2020-10-22

**Authors:** Lena Cohen, Irit Sagi, Einat Bigelman, Inna Solomonov, Anna Aloshin, Jeremy Ben-Shoshan, Metsada Pasmanik-Chor, Zach Rozenbaum, Gad Keren, Michal Entin-Meer

Metsada Pasmanik-Chor is not included in the author byline. Metsada Pasmanik-Chor should be listed as the seventh author and affiliated with 4: Bioinformatics Unit, Faculty of Life Sciences, Tel-Aviv University, Tel-Aviv, Israel. The contributions of this author are as follows: Formal Analysis, Methodology, Software, Writing–Review and Editing.
