# Supplementary figures and images for: Cardiac remodeling secondary to chronic volume overload is attenuated by a novel MMP9/2 blocking antibody
Source: PLoS One. 2020 Apr 9;15(4):e0231202. doi: 10.1371/journal.pone.0231202 (PMC7145114; doi:10.1371/journal.pone.0231202)

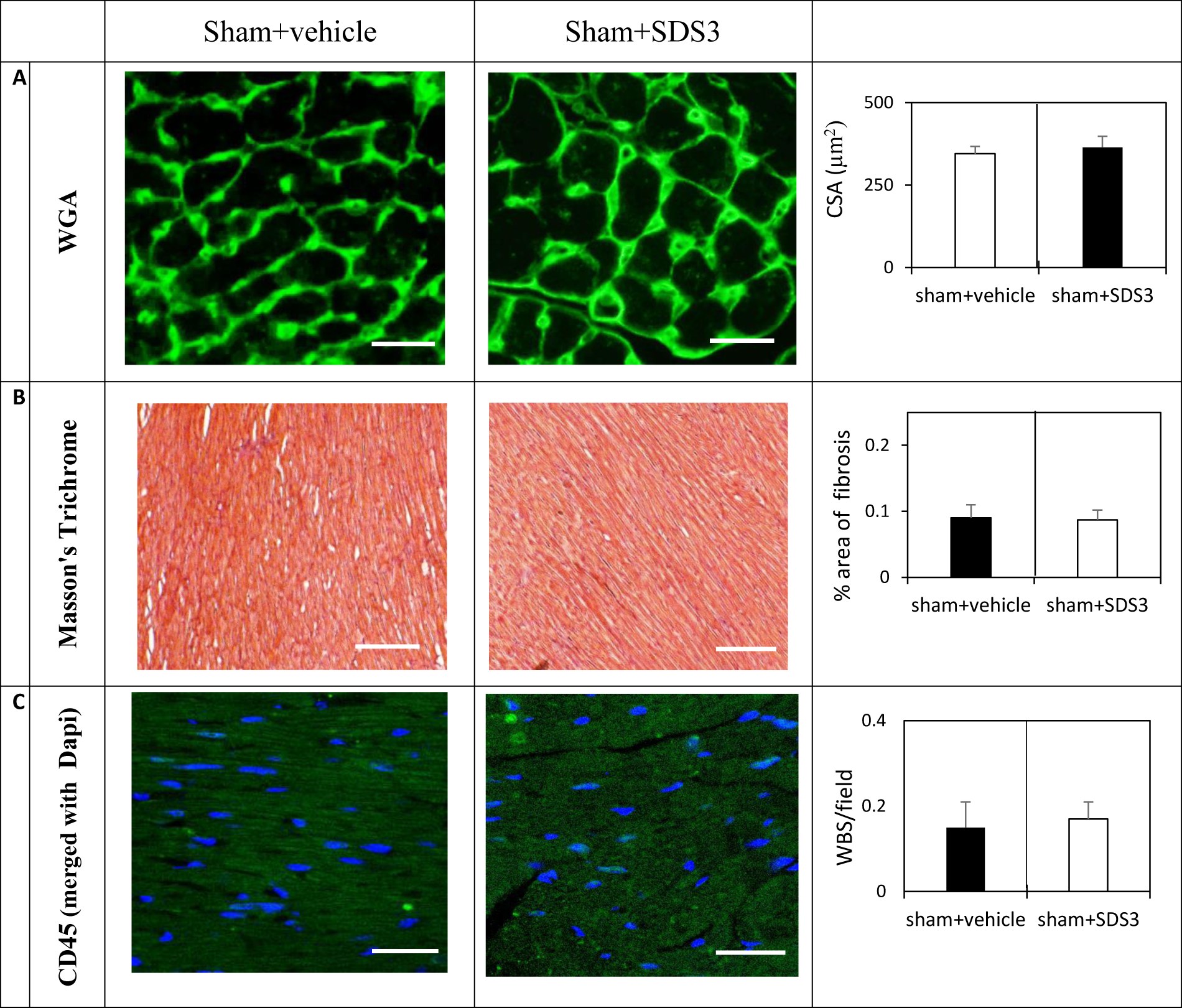

Supplement: S1 Fig — (A) Left panel: representative pictures illustrating LV myocyte cross-sections stained with WGA. Right panel: cross-sectional area (CSA) quantification (n = 3-4/group). Scale bar—100 μm (B) Left panel: Representative Masson's trichrome-stained histological sections; Right panel: Percentage of fibrosis quantification (n = 5/per group). (C) Left panel: Representative immunofluorescent images of CD45 expression (green) in LV sections. Dapi staining of nuclei is represented in blue. Right panel: Mean total number of white blood cells per field. Scale bar -25μm. (TIF) [file pone.0231202.s001.tif]
