# Supplementary material for: Cardiac remodeling secondary to chronic volume overload is attenuated by a novel MMP9/2 blocking antibody
Source: PLoS One. 2020 Apr 9;15(4):e0231202. doi: 10.1371/journal.pone.0231202 (PMC7145114; doi:10.1371/journal.pone.0231202)
Supplement: S1 Data — (PDF) [file pone.0231202.s002.pdf]

S1. Primer sequences used for RT-PCR

MMP9: sense: CAGACGTGGGTCGATTCC

antisense: TCATCGATCATGTCTCGC

MMP2: sense: ACGATGATGACCGGAAGT

antisense: GTGTAGATCGGGGCCATC

The relative mRNA expression was normalized to the expression of the GAPDH reference gene:

GAPDH: sense: TGTGTCCGTCGTGGATCTGA

antisense: TTGCTGTTGAAGTCGCAGGAG
